# Exosome microRNAs in Metabolic Syndrome as Tools for the Early Monitoring of Diabetes and Possible Therapeutic Options

**DOI:** 10.3390/ph14121257

**Published:** 2021-12-02

**Authors:** Erika Cione, Roberto Cannataro, Luca Gallelli, Giovambattista De Sarro, Maria Cristina Caroleo

**Affiliations:** 1 Department of Pharmacy, Health and Nutritional Sciences, Department of Excellence 2018-2022, University of Calabria, Ed. Polifunzionale, Arcavacata di Rende, 87036 Rende, CS, Italy; mariacristinacaroleo@virgilio.it; 2 GalaScreen Laboratories, University of Calabria, Ed. Polifunzionale, Arcavacata di Rende, 87036 Rende, CS, Italy; rcannataro@nutrics.it; 3 Department of Health Science, University of Catanzaro and Operative Unit of Clinical Pharmacology and Pharmacovigilance, Mater Domini Hospital, 88100 Catanzaro, CZ, Italy; gallelli@unicz.it (L.G.); desarro@unicz.it (G.D.S.)

**Keywords:** diabetes, exosomes, insulin resistance, miRNAs, therapy

## Abstract

Exosomes are nano-sized extracellular vesicles produced and released by almost all cell types. They play an essential role in cell–cell communications by delivering cellular bioactive compounds such as functional proteins, metabolites, and nucleic acids, including microRNA, to recipient cells. Thus, they are involved in various physio-pathological conditions. Exosome-miRNAs are associated with numerous diseases, including type 2 diabetes, a complex multifactorial metabolic disorder linked to obesity. In addition, exosome-miRNAs are emerging as essential regulators in the progression of diabetes, principally for pancreatic β-cell injury and insulin resistance. Here, we have clustered the recent findings concerning exosome-miRNAs associated with β-cell dysfunction to provide a novel approach for the early diagnosis and therapy of diabetes.

## 1. Introduction

Diabetes is a metabolic disorder stemming from defective insulin secretion and the occurrence of insulin resistance in peripheral tissues. Obesity, dietary fat intake, and physical inactivity are recognized as the main risk factors [1,2]. The close link between obesity and the development of this metabolic disorder have led to the creation of the new term “diabesity”, which combines the burden of obesity and diabetes [3]. This condition will affect more than 650 million people by 2045, with a concomitant increase in sanitary health costs concerning diabesity-related diseases [4,5,6]. Despite the considerable advancement in the understanding and treatment of diabetes, the correlated morbidity and mortality rates have continued to increase. Therefore, there is an urgent need for biomarkers to improve the clinical diagnostic process and the therapeutic approach of diabetes [7].

A number of extracellular vesicles (EVs) exist in all human fluids [8,9]. Their classification is based on their size: (i) large EVs (diameter > 200 nm) and (ii) small EVs (diameter < 200 nm) of which exosomes (30–150 nm) belong [10,11]. Exosomes are broadly present in human body fluids such as cerebrospinal fluid, urine, semen, saliva, and breast milk [12,13,14]. Exosomes can carry bioactive molecules and are essential for cell–cell communication [15,16].

With the conclusion of the Human Genome Project and the opening of the postgenomic era, non-coding RNAs (ncRNAs) have gained attention in numerous research fields [17,18,19]. miRNAs are a type of ncRNA with approximately 22 nucleotides encoded by endogenous genes [20]. They act as regulators of post-transcriptional gene expression by directing target mRNA cleavage or translational inhibition. More than one-third of human genes are thought to be regulated by miRNAs, revealing their involvement in various physiological and pathological processes. miRNAs are tissue-specific and more stable as compared with long non-coding RNAs (ln-RNAs) and messenger-RNAs (mRNAs) because of their shorter sequences [21,22]. miRNAs can be packaged within exosomes, which deliver and release them into target tissue cells. Of note, approximately 100 miRNAs have been identified in the exosomes produced by mast cells [23,24]. Exosome-miRNAs participate in normal physiological processes and are also involved in the occurrence and development of several diseases [25,26,27]. In this frame, they are emerging as crucial regulators in the onset and development of diabetes. Moreover, exosome-miRNAs released into systemic circulation can be used as diabetes markers because of their specificity and sensitivity [28,29]. In this review, we provide an overview on the role of exosome-miRNA-mediated mechanisms in the development of diabetes as established to date, outlining a hypothetical basis for the potential use of exosome-miRNA as diabetes healing targets and/or as a possible therapy themselves.

## 2. Characteristics of Exosome-miRNAs

The first observation of exosomes was by Trams et al. in 1981, who detected “*small membranous vesicles in the supernatants of tumor cells cultured in vitro*”. Those macrovesicles were called exosomes [30]. At that moment, it was believed that the function of exosomes was only for the waste disposal system for cells. Instead, further research has highlighted exosomes’ role in several biological processes encompassing the immune response, cell differentiation, and cancer [31,32]. Exosomes are a subtype of extracellular vesicles that can be identified based on their endosomal origin and their size, which ranges from 30 to 150 nm. Their biogenesis initiates with the formation of early endosomes by the inward budding of the cell membrane followed by the second inward budding of the endosomal membrane. The second inward budding results in the formation of late endosome (intraluminal vesicles). Late endosomes comprising intraluminal vesicles (ILVs) are identified as multivesicular bodies (MVBs). During the maturation phase from early endosome to MVBs, the cargoes are incorporated into ILVs through endosomal-sorting complex-dependent or endosomal-sorting complex-independent pathways. MVBs can be transported to the trans-Golgi network for endosome recycling, delivered to lysosomes for degradation, or move along microtubules to fuse with the plasma membrane and release exosomes into the extracellular space. MVB fusion with the cellular membrane is a fine-tuned process, which requires several crucial factors. Exosomal cargoes from the source cell can be further delivered to target cells via endocytosis, direct membrane fusion or receptor–ligand interactions [31]. Almost all mammalian cells produce and release exosomes, including the blood cells: (i) B lymphocytes, (ii) T lymphocytes, (iii) platelets, (iv) mast cells, and (v) dendritic cells, but also: epithelial cells, astrocytes, and neurons [33,34,35,36,37,38,39]. Exosomes have been reported in all biological fluids, and their composition reflects the metabolic state of the cell of origin. Of note, exosome can be selectively taken up by neighboring or distant cells far from their release, reprogramming the functional activity of the recipient cells through the delivery of bioactive molecules. Thus, exosomes and their biologically active cargoes may offer potential biomarkers of diagnosis and therapeutic targets in a range of diseases, such as chronic inflammation, cardiovascular and neurodegenerative diseases, cancer, obesity, and metabolic diseases [34]. In addition to specific proteins, exosomes also contain different patterns of RNAs that can be delivered to recipient cells. RNA sequencing analysis demonstrated that miRNAs were the most abundant in human plasma-derived exosomal RNA species [40]. Exosomes-miRNAs undergo unidirectional transfer between cells, leading to an intercellular trafficking network. The latter elicits transient or persistent phenotypic changes in recipient cells [41]. It was proven that after entering a target cell, miRNA released from the exosome can interact with the 3′-UTR region of the targeted mRNA, resulting in inhibition of the specific gene expression [42]. It is worth mentioning that in addition to miRNAs, long RNA species, especially long non-coding RNAs and circular RNAs, have recently been reported to exist in exosomes and affect a variety of biological processes, including the development of cancer [43].

Exosome-miRNAs circulating in body fluid can also act as biomarkers to mirror disease progression. Gathering evidence indicates that exosome-miRNAs are essential in developing diseases; therefore, their use as biomarkers for disease diagnosis, prognosis, and personalized therapy is becoming more apparent [44,45].

## 3. Dysregulation of Exosome-miRNAs in Diabetes

**Figure 1:**
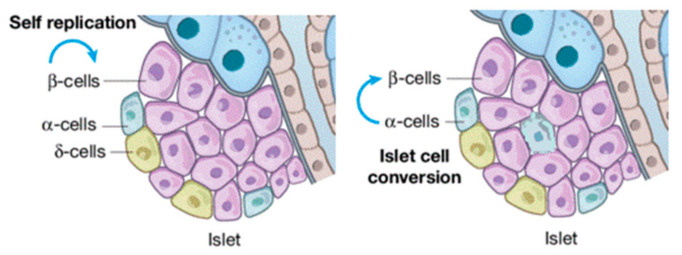
β-cell injuries reshape islet cellular assets.

The continuous increase in diabetes prevalence and incidence renders this metabolic disorder a global public health emergency [5]. Diabetes can be categorized into: (i) type 1 diabetes, (ii) type 2 diabetes, also known as alimentary diabetes, and (iii) gestational diabetes (genetic types are rare). Chronic hyperglycemia reshapes islet cellular assets with the infiltration of α-cells in the core of β (Figure 1). More serious is the complication that long-term hyperglycemia does in the damage, dysfunction, and failure of multiple organs, particularly blood vessels, nerves, kidneys, heart, and eyes [46,47]. Therefore, consequences that can be recognized as diabetic are: (i) retinopathy, (ii) macro-vascular complications, (iii) nephropathy, (iv) cardiomyopathy, and (v) foot ulcers [48]. Diabetes-related morbidity and mortality can be reduced by the improvement of preventive care, early clinical diagnosis, and appropriate therapeutic approaches [48,49]. Hence, identifying effective biomarkers to prevent and treat diabetes earlier, as well as its complications, are needed. Since we are in the precision medicine era, increasing attention is being paid to diagnosing and treating diseases [17,30]. In this frame, exosomes are a useful tool for the early diagnosis and treatment of diseases, including diabetes [18,19,31,32]. Furthermore, several miRNAs are being identified in β-cell dysfunction and miRNAs, such as let-7, miR-29, miR-223, and miR-103, are able to control metabolism in disorders such as diabetes. Their modulatory effects involve multiple pathways, spanning from liver metabolism to the fine tuning of insulin secretion [50,51,52]. Protected exosomes-miRNAs also play a critical role in diabetes development and progression, and its associated complications which mostly yield pancreatic β-cell injury and insulin resistance [53,54,55,56,57,58].

## 4. Mechanism of Exosome-miRNAs in Diabetes Progression

**Figure 2:**
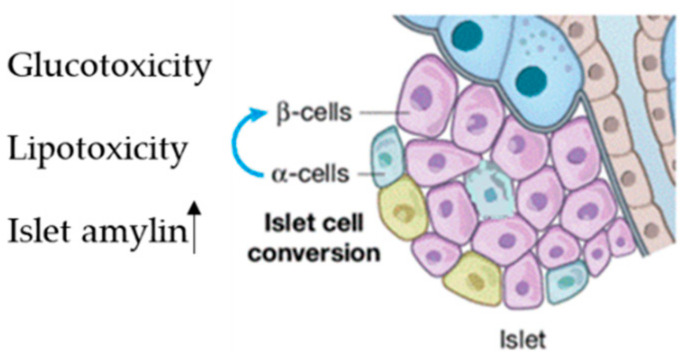
Factor inducing islet reshaping, Lipo and glucotoxicity and increasing of islet amylin.

About 70% of pancreatic cells are β-cells, which play a fundamental role in sustaining blood glucose homeostasis via insulin secretion into systemic blood circulation [59]. β-dysfunction due to cell injury leads to the progression of diabetes [60]. This occurs in the early pre-diabetes stage and is characterized by three main mechanisms: (i) the first is hyperglycemia; (ii) the second is elevated free fatty acid levels; (iii) the third is high amylin levels, which is co-secreted with insulin and induces β-cell apoptosis [59] (Figure 2).

**Figure 3:**
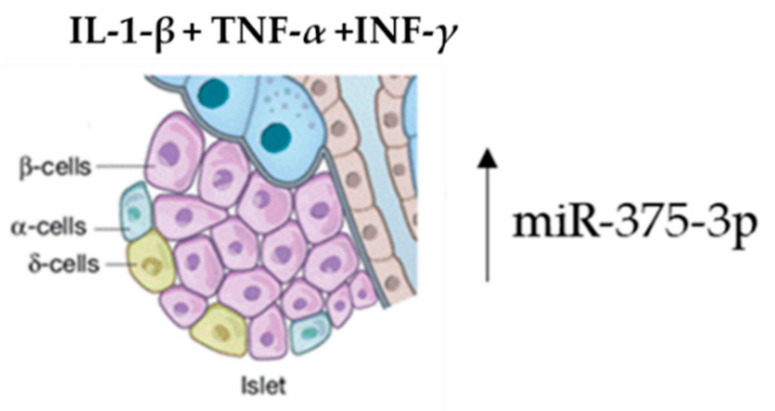
Acute β-cell injury using a mixture of cytokines induces an elevation of miR-375-3p.

Several studies have proved that the enrichment of specific exosomal miRNAs can target genes having an essential conservation outcome on pancreatic β-cell function in the initial stages of diabetes. Both high glucose and fatty acid levels negatively regulate this pattern, determining an enrichment of exosome-specific miRNAs involved in β-cell dysfunction in diabetes [61,62,63]. Using an ICR mouse model, Fu et al. demonstrated that the concomitant administration of interleukin-1-beta (IL-1-β), tumor necrosis factor-alpha (TNF-α), and interferon-gamma (INF-γ) induced β-cell injury [64]. Islet tissue isolation from these mice and exosome-miRNA revealed a significant change in the miR-375-3p expression levels. Furthermore, this microRNA was also found to be higher in diabetes patients versus normoglycemic patients. Therefore, hsa-miR-375-3p could be considered as an early marker of islet injury (Figure 3).

The pool of exosome-miRNAs deriving from other cells can act on β-cells. This was demonstrated by treating MIN6B1 pancreatic cells with a mixture of IL-1-β, TNF-α and INF-γ cytokines. The exosome enrichment containing miRNAs, secreted in the medium, can be delivered to contiguous β-cells, inducing cell death [54].

**Figure 4:**
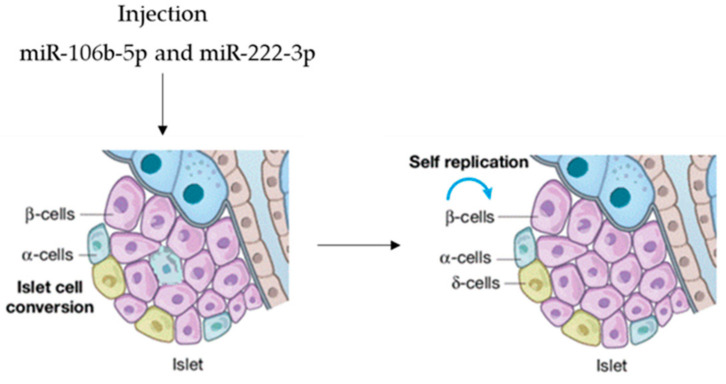
microRNAs as therapeutic option.

Tsukita et al. screened miRNA levels, pointing out significant changes in the serum exosomes of mice after bone marrow transplantation [65]. Forty-two miRNAs were upregulated after bone marrow transplantation, and of these, miR-106b-5p and miR-222-3p were released by bone marrow cells and transported to pancreatic islet cells, inducing β-cell renewal. In view of possible microRNA-based therapy, agomir miR-106b-5p and miR-222-3p were tail-vein-injected into mice, promoting the proliferation of injured β-cells. It was demonstrated that the injection of both miRNAs leads to the downregulation of the Cip/Kip family, which, in turn, improves hyperglycemia in insulin-deficient diabetes mice. This is evidence that they can function as a therapeutic option to rescue from β-cell dysfunction (Figure 4), so that circulating miRNAs are endocrine factors that facilitate metabolic organ crosstalk.

Exosome-miRNAs secreted by β-cells can be transferred to other acceptor tissue cells which in turn regulates β-cell activity. For example, when exosome miR-26a [66] is transferred to the liver, it improves the insulin sensitivity of the acceptor cells, maintaining metabolic homeostasis. In addition, serum miR-204 is strictly associated with pancreatic β-cell injury, which could be helpful as a novel biomarker for early type 1 diabetes [67]. The findings indicate how exosome-miRNAs are strictly related to β-cell damage and dysfunction in diabetes.

### Exosome-miRNAs and Insulin Resistance

Insulin resistance is a pathological condition in which target cells or tissues show reduced sensitivity or reactivity to insulin. It is a distinct feature of diabetes. Under this condition, the physiological levels of the hormone fail to preserve normal glucose homeostasis. Insulin resistance mainly occurs in fat, muscle, and liver cell populations, which use insulin to facilitate glucose uptake [68]. The condition is also characterized by the impairment of insulin signaling encompassing the insulin receptor, the insulin receptor substrate 1/2 (IRS-1/2), the glucose transporter 4 (GLUT4), and the phosphoinositide 3-kinase (PI3K)/AKT serine/threonine kinase (AKT) [69,70,71,72]. The mechanism underlying insulin resistance involves oxidative stress, inflammation, and autophagy. Recently, it has been shown that miRNAs in exosomes contribute to the mechanism of insulin resistance [73,74,75,76,77,78] and high expression of miR-20b-5p in exosomes from diabetic patients was also found. This miRNA modulates glucose metabolism in human skeletal muscle cells through AKT signaling, thus regulating the incidence of insulin resistance [79]. In agreement with these data, a further study pointed out that pancreatic-cancer-derived exosomes miR-450b-3p and miR-151-3p entering in myoblast C2C12 mouse cells can inhibit PI3K/AKT signaling. This mechanism inhibits Glut4 transport and sustains insulin-induced FoxO1 rejection [80]. Furthermore, it was demonstrated that exosomes miR-27a and miR-320a are correlated with metabolic syndrome and diabetes. Exosomes miR-23a, miR-197, and miR-509-5p are linked to dyslipidemia [81]. Exosome-miRNAs are strictly related to aging insulin resistance. In this frame, the involvement of exosome miR-29b-3p from bone marrow mesenchymal stem cells has been reported and its potential role as a therapeutic tool in senescence-induced insulin resistance has also been suggested. Obesity is a further high-risk factor in the occurrence of insulin resistance. Obesity deeply affects the expression profile of plasma exosome-miRNAs in mice and humans [82,83]. In mice, it was reported that the expression of plasma exosome-miRNAs in obese mice compared with lean mice, including miR-27a-3p, miR-27b-3p, miR-122, and miR-192, was increased. Furthermore, glucose tolerance was induced in lean mice exposed to exosomes isolated from obese mice [84]. In line with this evidence, Ying et al. showed that adipose tissue macrophages in obese mice secrete miRNA-containing exosomes, which cause glucose intolerance and insulin resistance when administered to lean mice. The opposite is also true. Altogether, these findings unveil the pivotal role of exosome miRNAs in the pathogenetic mechanisms of insulin resistance [85]. microRNAs involved in metabolic syndrome are summarized in Table 1.

## 5. Exosome-miRNAs in Diabetes: Potential Clinical Applications

Given that diabetes belongs to chronic metabolic disorder diseases, the early monitoring of glycemia, blood pressure, and cardiovascular risk can improve the potential therapeutic effect of exosome-miRNAs in diabetes patients [7]. Owing to their peculiar structure, exosome-miRNAs are more stable in tissues and cells and can be specifically detected using qRT-PCR or in situ hybridization assay [86]. In addition, they are developmentally regulated [87]. Recent studies have shown that the profile of exosome-miRNAs affects the outcome of diabetes and its associated complications. Further evidence revealed that the exosome miRNAs’ expression profile in sera and urine differs between healthy and diabetic individuals. These findings strongly suggest the potential role of exosome-miRNAs as novel diagnostic biomarkers of diabetes [87,88,89,90,91,92,93,94,95]. In this conceptual framework, miR-1, miR-133a, miR-30a, miR-342, and miR-133b are most promising candidates [88,93]. Furthermore, Sidorkiewicz et al. [94] showed that exosome-miR-491-5p, miR-1307-3p, and miR-298 could be helpful biomarkers in monitoring the progression of diabetes. It is worth mentioning that exosome-miRNAs are closely associated with the gender difference of diabetes, as reported by Deng and co-workers [95]. They found that serum miR-29a/b levels were decreased in diabetic pregnant women and correlated with neonatal pathologic jaundice, showing a diagnostic value for these miRNAs. Exosome-miRNAs cannot only be used as biomarkers, but can also serve as miRNA inhibitors and agonist delivery systems for the treatment of diabetes. In this frame, the widespread application of nanotechnology has fostered the use of exosome-miRNAs in animal models. In addition, several engineered exosome-miRNAs have been developed to accelerate wound healing in diabetic rats, taking advantage of the natural availability and biocompatibility of cellular-derived exosomes as extracellular miRNA-transporting particles. The engineered exosomes exhibited excellent effects on re-epithelization, vessel maturation, angiogenesis, and collagen remodeling, leading to a novel therapeutic strategy in diabetic chronic wound healing [96]. Of note, Shi et al. [97], using diabetic pregnant mouse models, demonstrated that maternal exosomes in diabetes could cross the maternal–fetal barrier and contribute to cardiac development deficiency via miRNAs, providing new insights into chronic heart disease prevention and treatment. Studies on exosome inhibitors or agonists suggest a novel strategy for mitigating exosome-mediated diabetes and associated complications. However, the translation of experimental findings into clinical practice remains challenging, as well as the use of exosome-miRNAs for diabetes treatment. Further research focused on biomaterials and on the function and mechanism of exosome-miRNAs could lead to innovative strategies for the clinical management of diabetes.

## 6. Challenging Tasks and Opportunities of Exosome miRNAs in Diabetes

The rapid evolution of next-generation sequencing technologies has led to the discovery and identification of an increasing number of exosome miRNAs. Recently, several databases have been developed to identify and predict exosome components (proteins, miRNAs, mRNAs, etc., and lipids) [98,99,100,101,102,103,104,105,106,107,108,109,110,111,112]. Of them, databases such as CMEP, Xeno-miRNet, and Mirandola can be utilized to highlight disease-related exosome miRNAs. The combination of biological sciences and material technology has also prompted the use of nanotechnology in the field of disease treatment [113,114], including diabetes [115]. Differently from the direct administration into the target tissues of the agomir or antagomir, nanocarrier-derived miRNAs displayed higher efficiency and specificity; the use of exosomes as endogenous nanocarriers has several advantages, including multiple drug loading, lack of toxicity, protection from drug degradation, and the delivery of drug cargoes to the disease-associated targeted cells [116]. Regarding exosome-miRNAs’ pivotal role in the progression of diabetes and its associated co-morbidities, the use of exosomes as endogenous nanocarriers has significant potential as a biological tool for diagnosing or treating diabetes.

## 7. Conclusions

Exosome-miRNAs have been recognized as powerful tools for integrating the diagnosis and treatment of diabetes. As regulatory molecules, they are involved in multiple steps of diabetes by modulating the expression levels of related genes. Furthermore, the possibility of silencing or activating exosome-miRNAs exogenously by incorporating antagomir or agomir into exosomes, followed by their injection in target tissues, has set the fundamentals for a new therapeutic approach in the treatment of diabetes. In any case, much remains to be done before the research findings might be translated into clinical practice. The future of exosome therapy includes combinations of targeted exosomes with antidiabetic drugs and/or exosomes as micronutrients [117], as well as high-precision diabetes diagnostic probes to create multifunctional platforms for in vivo tracking, prognosis monitoring, and therapy. Similarly, the first mRNA-based severe acute respiratory syndrome coronavirus 2 (SARS-CoV-2) vaccine encapsulated in lipid nanoparticles mimic exosome now exists [118].

## Figures and Tables

**Table 1:** Exosome-miRNA involved in the metabolic syndrome. For each miRNA, the cell of origin and the site of action are reported. BM-MSC, bone marrow mesenchymal stem cells.

Exosome-miRNA	Cell Type Origin	Action Site	References
Let-7	Immune cells, Endothelial cells	Liver cells, Pancreatic β-cells	[50]
miR-29	Pancreatic β-cells, Adipocytes, Hepatocytes	Liver cells, Pancreatic β-cells	[52]
miR-223	Pre-adipocytes, Adipocytes, Macrophages	Liver cells, Pancreatic β-cells	[51]
miR-103	Adipocytes, BM-derived stromal cells	Liver cells, Pancreatic β-cells	[53]
miR-375-3p	Pancreatic β-cells	Pancreatic β-cells	[63,64]
miR-106b-5p	BM-MSC	Pancreatic β-cells	[65]
miR-222-3p	BM-MSC	Pancreatic β-cells	[65]
miR-26a	Adipocytes, Hepatocytes, Vascular endothelial cells	Pancreatic β-cells	[66]
miR-20b-5p	Adipocytes, Hepatocytes	Skeletal muscle cells	[79]
miR-27a/b-3p	Endothelial cells, Adipocytes, Hepatocytes Glomerular mesangial cells	Liver cells Adipocytes	[82,83]
miR-320a	Adipocytes, Hepatocytes, Macrophages, Neutrophils	Liver cells, Adipocytes, Myocytes	[81,82]
miR-23a	Macrophages, Endothelial cells, Adipocytes	Liver cells	[81]
miR-197	Endothelial cells, Adypocytes	Liver cells	[81]
miR-509-3p	Adipocytes, Macrophages	Liver cells	[81]
miR-29b-3p	BM-MSC	Liver cells, Adipocytes	[84,85]
miR-122	Hepatocytes	Liver cells, Adipocytes	[84,85]
miR-192	Hepatocytes	Liver cells, Adipocytes	[84,85]
